# How do the existing homecare services correspond with the preferred service ecosystem for senior citizens living at home? A qualitative interview study with multiple stakeholders

**DOI:** 10.3389/frhs.2024.1294320

**Published:** 2024-03-21

**Authors:** Christophe Eward Kattouw, Karina Aase, Petter Viksveen

**Affiliations:** SHARE—Centre for Resilience in Healthcare, Department of Quality and Health Technology, Faculty of Health Sciences, University of Stavanger, Stavanger, Norway

**Keywords:** service ecosystem, senior citizens, stakeholder involvement, patient-centered care, home care services, health services research, qualitative research, idealized design approach

## Abstract

**Introduction:**

Often, homecare services are task-focused rather than person-based and fragmented instead of integrated. Consequently, several stakeholders have requested a transformation of the service ecosystem for senior citizens living at home. This transformation may be facilitated by an idealized design approach. However, few studies have applied such an approach. Moreover, previous research did not assess the ways in which the existing homecare services correspond with the preferred service ecosystem for senior citizens living at home. Therefore, the purpose of this study is to gain an understanding of how the existing homecare services correspond with the preferred service ecosystem for senior citizens living at home, according to different stakeholders.

**Methods:**

Four stakeholder groups (*n* = 57) from a Norwegian municipality participated in an interview study (2019–2020): senior citizens, carers, healthcare professionals and managers. A directed qualitative content analysis was applied, guided by a four-category framework for the preferred service ecosystem.

**Results:**

All stakeholder groups highlighted several limitations that hindered continuity of the services. There was also agreement on deficiencies in professionals’ competence, yet professionals themselves did not focus on this as a significant aspect. Managers emphasised the importance of professionals’ reablement competence, which was also considered to be deficient in the current homecare services. Contrary to the other stakeholder groups, most senior citizens seemed satisfied with the practical and social support they received. Together with carers, they also explained why they thought some professionals lack compassion. Their dependency on professionals may limit them in sharing honestly their opinions and preferences during care provision. Involvement of senior citizens in improvement of the current services was limited. Insufficient time and resources, as well as a complex organisation impacted the existing homecare services, and therefore served as barriers to the preferred service ecosystem.

**Discussion:**

In this study there were different degrees of correspondence between the existing homecare services and the preferred service ecosystem according to four stakeholder groups. To develop the preferred service ecosystem, aspects such as predictability, adaptivity, and relationships are key, as well as continuous involvement of senior citizens and other stakeholders. The four-category framework applied in this study served as a tool to assess the existing homecare services.

## Introduction

Most senior citizens want to live independently at home as long as possible and remain social and active within the community (1–4). Due to growing health challenges and care needs, national and municipal policymakers increasingly emphasise the provision of homecare services for senior citizens (5–9). Some of the service models are based on person-centred care, but evidence state that they are challenging to implement, or lack the emphasis on age-friendly components and infrastructures (2, 10–12). Other homecare service models are task-focused rather than person-focused, struggle to adapt to changing needs, and in many instances appear as fragmented instead of integrated (5, 13–22). Hence, they pose a threat to senior citizens’ dignity and independence. Existing service delivery models in home care experience these challenges both globally and in the Scandinavian countries (5, 13–22). Consequently, various stakeholders have requested a transformation of the service ecosystem for senior citizens living at home, based on what matters to them (11, 15, 23–26). A service ecosystem can be understood as “a relatively self-contained, self-adjusting system of resource-integrating actors connected by shared institutional arrangements and mutual value creation through service exchange” (27). This implies value co-creation by multiple stakeholders, who may have distinctive views on institutional arrangements such as meanings, norms and rules (28–33). An idealized design approach may facilitate the transformation of the preferred service ecosystem for senior citizens living at home. Such an approach implies first exploring the characteristics of the desired service ecosystem from multiple stakeholders’ perspectives. Subsequently, a comparison between an idealized service design and the existing design could facilitate the identification of service characteristics which can be preserved, and what needs to be improved or redesigned. In other words, this comparison could identify the gaps that need to be closed (34–36).

To date, research applying an idealized design approach is scarce. Moreover, we have been unable to identify any other studies assessing the correspondence and discrepancies between the current homecare services and the preferred service ecosystem. The aim of this study is therefore to gain an understanding of how the existing homecare services correspond with the preferred service ecosystem for senior citizens living at home, according to different stakeholders: senior citizens, carers, healthcare professionals and managers. The study is a part of a research project aiming to develop design proposals for the preferred service ecosystem for senior citizens living at home in Norway.

In Norway, most homecare services are public and the government delegates service provision to the 365 municipalities. The municipalities are free to organise the homecare services as they see fit, provided they meet the requirements for safe and sound care. While community nursing should be free of charge, additional services like practical assistance (e.g., home cleaning) and access to senior citizen centres are usually not free of charge. In many municipalities, reablement teams support senior citizens temporarily to live at home as long as possible. In most municipalities, resource allocation offices make care decisions concerning the type, duration and frequency of homecare services (5, 37).

## Materials and methods

A qualitative design (38) was applied, with focus groups and semi-structured individual interviews (39, 40). Individual interviews were conducted with senior citizens who for health-related reasons were unable to participate in focus group interviews. Managers were interviewed individually in order to prevent potential power differentials which might arise when including them in focus group interviews with other stakeholder groups. The individual and focus group interviews provided insight into the perspectives of 57 senior citizens, carers, professionals and managers on how the existing homecare services correspond with the preferred or “ideal” service ecosystem. The homecare service characteristics of the municipality included in this study are presented in Table 1 and the study participants in Table 2. The consolidated criteria for reporting qualitative research (COREQ) checklist was used to report this study (41). Moreover, the central principle of stakeholder involvement was used throughout the entire study, in order to gain insights from senior citizens, carers, healthcare professionals and managers on how the existing homecare services correspond with the preferred service ecosystem for senior citizens living at home.

**Table 1:** Characteristics of the municipal homecare services (during data collection).

Nursing teams (*n* = 9)
Professionals per nursing team: M = 52 (range 41–66)
Untrained assistants: M = 19 (range 10–33)
Nurses: M = 10 (range 6–12)
Skilled healthcare workers: M = 9 (range 7–21)
Social workers: numbers not available
Senior citizen centres (*n* = 7)
Reablement team (*n* = 1): physiotherapists, occupational therapists, nurses and skilled health workers

**Table 2:** Interview and participant characteristics.

Stakeholder group type/interview type^a^	Interview participants (*n* = 82)^b^	Participant characteristics
Senior citizens (*n* = 26)^c^	34	Age 71–90 (M = 83), female *n* = 18, male *n* = 8
Focus group 1	11	Focus groups: 42% received community nursing, Individual interviews: all received community nursing daily
Focus group 2	7	
Focus group 3	6	
Focus group 4	5	
Focus group 5	5	
Individual 1–5	5	
Carers (*n* = 4)^c^	5	Age 74–80 (M = 77), female *n* = 2, male *n* = 2
Focus group 6	4	Spouses received community nursing
Individual 6	1	
Healthcare professionals (*n* = 22)^c^	28	Female *n* = 21, male *n* = 1
Focus group 7	8	Professional background: nurses *n* = 10, skilled health worker *n* = 8, physiotherapist *n* = 2, occupational therapist *n* = 1, social worker *n* = 1
Focus group 8	6	
Focus group 9	10	
Focus group 10	4	
Managers (*n* = 5)^c^	10	Female *n* = 5
Individual 7–16	10	Nurses *n* = 4, physiotherapist *n* = 1

^a^ Individual interviews *n* = 16, focus group interviews *n* = 10.

^b^ 29 participants were interviewed more than once (explained in the section “data collection procedures”).

^c^ Number of unique participants for each stakeholder group, i.e., those who were interviewed more than once were only counted once.

### Setting

The current study was conducted in one of Norway's 20 largest municipalities (>75,000 inhabitants), including urban and rural areas.

### Recruitment and sample

Three user organisation representatives, and municipal homecare service managers actively participated during the planning phase, to enhance the study's relevance and feasibility, and to provide recommendations for effective and user-friendly recruitment of participants.

These representatives and managers contributed valuable recommendations to determine the sample selection, interview guides, and recruitment protocols. For example, user organisation representatives recommended including carers in the study.

Inclusion criteria for the study were: (a) senior citizens 67 or older with homecare experience, (b) carers of senior citizens receiving homecare services, (c) healthcare professionals, and (d) managers from the different municipal homecare service teams (Table 1). Participant recruitment was facilitated by the municipal dementia coordinator and the managers. In line with the study aim, they informed potential participants across the municipal homecare service teams, and provided them with written information about the research project. The senior citizens and carers who participated in individual or focus group interviews were approached by managers or the municipal dementia coordinator. All participants who were approached agreed to take part in the study. However, eight professionals were unable to attend the second interview due to sick leave or misunderstanding about the time of the interview. The second focus group interview with carers had to be cancelled due to Covid-19 restrictions. Instead, an individual interview was conducted in one carer's home. The carers attending the focus group in the first stage also shared their perspectives on the existing homecare services. A total of 57 participants were involved in this study, including 30 senior citizens and carers, and 27 healthcare professionals and managers (Table 2).

### Data collection procedures

Data was collected in two stages in 2019 and 2020 through 16 individual and 10 focus group interviews. Most participants were interviewed twice. In the first interviews, participants were asked about their perspectives on the preferred service ecosystem (2). However, they also provided unprompted information about their experiences with the existing homecare services. The interviews carried out in the second stage focused explicitly on how the existing homecare services aligned with the preferred service ecosystem. Although data collection coincided with the onset of Covid-19, dialogue with all four stakeholder groups in 2023 did not suggest that any significant changes had been made to the existing homecare services. It was therefore decided that no additional data collection was required. Interview guides were tailored to the interview type (individual or focus group) and the stakeholder group (senior citizens, carers, professionals, or managers). Interview questions for senior citizens are presented in Table 3.

**Table 3:** Interview guide for senior citizens (stage 2).

Question A	Think about how you experience the existing homecare services. How or to what extent do you think that the existing homecare services cover what matters to you?
	Follow-up question: How or to what extent do you think that the existing homecare services support you in doing what you want?
Question B	With regard to what matters to you: What do you perceive as good in the existing homecare services?
	Follow-up question: Do you have examples that made you think: “This is the best experience I've had with the existing homecare services?"
Question C	With regard to what matters to you: What do you think could be improved in the existing homecare services?
	Follow-up question: Do you have examples that made you think: “This is the most challenging experience I've had with the existing homecare services?"
Question D^a^	D1: What do you think about the number of different healthcare professionals you are faced with?
	D2: How often do the professionals arrive on time?
	D3: How often do the professionals have sufficient time for you? (For example, so you can ask questions?)
	D4: To what extent do you remember the names of the professionals?
Question E	What do you think about technology that can help you living at home?

^a^ Questions addressing issues raised by stakeholders in stage 1.

The main questions presented for all stakeholder groups focused on a comparison of the existing homecare services with the preferred service ecosystem for senior citizens living at home (question A, B and C). Senior citizens and one of the carers were posed specific questions about the preferred service ecosystem as they brought up these issues in the interviews in the first stage of the research project. It addressed issues of the time professionals spent with senior citizens and the continuity of the services (questions D1–D4). Finally, user organisation representatives suggested that senior citizens should be asked about their perspectives on technology developed to support senior citizens living at home (question E).

During both individual and focus group interviews, participants were given one question at the time on a sheet of paper and a few minutes to reflect individually. Focus group participants were then asked to spend a few minutes to share their perspectives with each other (in pairs). This was followed by a plenary discussion. The process provided all participants with an opportunity to share their opinions and to further reflect on their experiences (42). All interviews were audio recorded (64–152 min, median 85) and transcribed verbatim.

### Data analysis

A directed qualitative content analysis (43) was carried out through six stages to analyse data according to a four-category framework for the preferred service ecosystem. The framework was developed in phase I of the research project (2). It was based on the four stakeholder groups’ perspectives and aims to meet senior citizens’ needs for self-reliance and remaining active and social within the community through: (1) support for living at home as long as possible; (2) accessible information and services; (3) continuity of services; and (4) compassionate and competent healthcare professionals (2). Tables 4, 5 provide a more detailed description of the framework's main- and sub-categories.

**Table 4:** Categories of the preferred service ecosystem (2).

Category	Description
Support for living at home as long as possible	Practical and social support provided by healthcare professionals is necessary, in particular if reablement and assistive devices are insufficient. This may alleviate family burden, prevent loneliness and despair. Actively involving senior citizens in their own care may ensure the relevance of services in meeting their needs.
Compassionate and competent health care professionals	Professionals who visit senior citizens in their homes should care about them, be friendly, offer support, and not take over tasks nor make decisions on behalf of senior citizens. They should take sufficient time and treat senior citizens with dignity. Furthermore, they should regularly update their competences, and expand their knowledge of senior citizens’ needs and preferences.
Continuity of services	Senior citizens’ needs, including social life needs, and biorhythms should determine the professionals’ visiting hours. A limited number of professionals working in smaller districts safeguards continuity of care and facilitates both interpersonal collaboration and “relationship building” with senior citizens and their carers.
Accessible information and services	Senior citizens should have easy access to information about available services, and assistive devices. In case of special needs senior, citizens should know who to contact and they should receive unambiguous answers. Senior citizen centres should have flexible opening hours depending on senior citizens’ and carers’ needs.

**Table 5:** How the existing homecare services correspond with the preferred service ecosystem.

Category	Sub–category	Senior citizens	Carers	Professionals	Managers
(1) Support for living at home as long as possible	*Practical and social support*	**++**	**− −**	**+/−**	**−**
	*Assistive devices*	**++**	**NA**	**− −**	**+/−**
	*Reablement*	**+/−**	**NA**	**NA**	**+/−**
	*Involvement*	**− −**	**NA**	**− −**	**+/−**
(2) Compassionate and competent health care professionals	*Professionals’ compassion*	**+**	**−**	**− −**	**+/−**
	*Professionals’ competence*	**− −**	**− −**	**NA**	**−**
(3) Continuity of services	*Timeliness and predictability*	**−**	**− −**	**− −**	**− −**
	*A limited number of professionals*	**−**	**−**	**− −**	**− −**
(4) Accessible information and services	*Information about services and access to professionals*	**+/−**	**− −**	**+**	**+**
	*Senior citizen centres*	**+**	**NA**	**+/−**	**−**

**^++^**Considerable correspondence, **^+^**Correspondence, **^+/−^**No clear tendency, **^−^**Discrepancies, **^− −^**Considerable discrepancies, **^NA^**Very few or no descriptions (explained in the section “data analysis”).

All interview transcripts were read several times by the first author, and 11 out of 26 interviews were assessed by the co-authors in order to gain an initial understanding of the perspectives presented by participants in each stakeholder group (stage 1). Initial insights were discussed by the three authors. All authors have extensive experience from clinical practice (CEK, PV) and research (CEK, KA, PV).

All participant quotes that contributed to the study's aim and research question were extracted and transferred to Excel (for Microsoft 365) by the first author (stage 2) to facilitate the directed content analysis. The co-authors checked a random selection of quotes to assess data extraction quality. The transcriptions were divided into meaning units, condensed (stage 3) and coded (stage 4). In stage 5, codes were categorized into the (sub)categories presented in Tables 4, 5. The sixth stage involved assessing and distributing codes according to whether they corresponded with the preferred service ecosystem or not. A degree of correspondence or discrepancy of more than 80% was classified to be considerable, from 60% to 80% was moderate, and less than 60% as unclear. Fewer than 15 codes were classified to be insufficiently described to be used and were therefore classified as “not applicable”.

Stages 2–6 were carried out by the first author and discussed and agreed with the co-authors. Supplementary File S1 exemplifies the analysis process. The results focus on the ways in which stakeholders’ description of the existing homecare services corresponded with or differed from their views of the preferred service eco-system for senior citizens living at home. Participant quotes were used throughout the text to illustrate the findings.

Several codes did initially not seem to fit with the established sub-categories. These codes addressed issues of time, resources and organisation of homecare services. The development of a new category, “time, resources and organisation”, was therefore considered. However, these codes were of importance to all four categories and are therefore presented throughout the results.

## Results

Interviews provided an insight into the perspectives of senior citizens, carers, healthcare professionals and managers on the existing homecare services and how these corresponded with their descriptions of the preferred service ecosystem (summarized in Table 5).

Table 5 shows the sub-categories and categories of the four-category framework. Furthermore, it shows alignments and discrepancies between the existing homecare services and the ideal service ecosystem from the perspectives of the different stakeholders. Insufficient time and resources, as well as a complex service organisation, significantly impacted the existing homecare services across all four categories, and therefore served as barriers to the preferred service ecosystem. These barriers are integrated into the results which are presented in the order given in Table 5.

### Support for living at home as long as possible

Contrary to the other stakeholder groups, senior citizens’ descriptions of the existing homecare services corresponded to a large extent with their views on the preferred service ecosystem to support them to live at home as long as possible. Most of all, their descriptions of the existing practical and social support, as well as the provision of assistive devices, were in line with their views of the preferred service ecosystem. In particular, it seemed that older senior citizens (80 + years) were pleased with the current services. Some of the younger senior citizens tried to explain differences of opinion between younger and older senior citizens:

Those who are 85 and approaching 90, they are more grateful for what they get. (Senior citizen 21 & 22) It’s because they are not used to asking for anything for themselves. (Senior citizen 22) They do not dare to say much. It’s like everything’s fine. (Senior citizen 20)

Another explanation might be that even though all senior citizens were reassured that stating their honest opinions during the interviews should not negatively influence the services they currently received, some may nevertheless have been concerned that it might have unwanted negative implications. One carer shared hints of such concerns when she described how she often withdraws from complaining about insufficient support and care:

I can't say that, because I have problems discussing such issues with professionals, because I am 100% dependent on having a good relationship with these people. Therefore, if I have something I need to discuss, I call the manager, and it’s quite rare that I do that as well. (Carer 2)

It could also be that professionals and managers are in a position to identify limitations of the existing homecare services due to their knowledge about service standards, regulation and legislation:

All the nice white papers, “guarantees”, big words. we see that it is not like this in reality. (Nurse 7)

Quite surprisingly, carers did not emphasise assistive devices, reablement and involvement in the existing homecare services (Table 5). Possibly, they perceived these aspects as less relevant as their spouses’ health condition was too poor. This was exemplified in the following statement:

We will never get back to where she was. It’s obvious that you cannot expect her to come back to how she was when she doesn’t remember when she was born. At one time things are okay, and then the opposite. […] They offered her reablement or training, but she refused. (Carer 4)

Even though some senior citizens and carers might not have fully described their dissatisfaction or concerns with the homecare services, it seemed clear that their awareness of the availability of the service served as a source of reassurance. The homecare services did also provide extensive practical support, such as delivering medicines to senior citizens’ homes and providing 24/7 care for those who were severely ill or in a terminal phase. Moreover, the professionals’ home visits served as an important source of social contact for many senior citizens, although visits were primarily scheduled to carry out practical tasks and the time for conversations was insufficient.

Particularly for the other stakeholder groups, there were considerable discrepancies between the existing services and the preferred service ecosystem regarding practical and social support, and involvement of senior citizens.

According to both professionals and managers, the existing homecare services were inadequate in meeting the senior citizens’ needs according to what was most important to them:

The way things are organized now determines what we can offer individuals, it is not always what they actually need or want. So I do not always feel that we take care of individual needs. It covers a social need and we reduce the burden for carers. But some support is difficult to provide, like shopping and cutting nails. (Nurse 4)

For example, assistance for showering was offered only once a week. Although some managers argued that this was mainly due to a lack of resources, it also seemed to be the result of an “old habit”:

I have been [working] in the homecare service for “a hundred years”, it has always been like that […] it has to do with the resources spent and what’s “good enough”, although I do not know who made that decision. (Manager 4).

Moreover, professionals provided insufficient support for household tasks and personal hygiene, leaving carers alone with these additional tasks:

The help they provide is good, but within a very limited area. We have to cut nails and manage foot care, wash hair, brush teeth, and I often have to wash his private parts because he smells of urine. He notices it and feels that it is unworthy. No one helps him at night to go to the bathroom, so it’s my job. (Carer 2)

Homecare services were also insufficient in meeting practical, physical, mental and social care needs. Both professionals and managers stressed that senior citizens were rarely accompanied by professionals to see their GP, and home visits by GPs were rarely carried out.

Professionals’ insufficient attention to senior citizens’ food and diet occasionally resulted in malnutrition and unhealthy weight loss. Additionally, professionals stated that neither time nor space was given for senior citizens to fulfil their sexual needs. Inadequate practical and social support was perceived as particularly critical for senior citizens with poor social networks, and psychosocial or cognitive impairment, for example due to dementia. Managers emphasised that it could be irresponsible for some senior citizens to continue living at home, but limited capacity precluded them from moving to a nursing home:

There are patients with dementia, where [professionals] do not feel it’s safe living on their own. Occasionally, it seems irresponsible to live at home. They started to leave the house on their own…using risky things like the fireplace. We cannot be there all the time, but then they should be somewhere else [nursing home]. (Manager 2)

Among all stakeholder groups, only managers explained service shortcomings by budget limitations and extensive resources spent for some groups of citizens, in particular those with mental health and drug problems.

Despite the need for outdoor social activities, local transport was according to senior citizens inadequate, in particular for those who struggled with mobility problems:

It became very difficult to take the bus with a walker. It’s about half a meter from the bus door to the curb. I need to lift the walker down at the edge of the curb, and up again and that’s too heavy for me. Now I don’t take the bus at all. I have heard several others say the same. (Senior citizen 24)

At the same time, senior citizens experienced that the existing assistive devices supported their ability to function at home and it provided a sense of safety. The latter promoted their independence and enabled them to live longer at home:

I easily stumble and break bones. I got an incline from the threshold down to the floor in the hallway, so it’s easier to get the walker up. […] They asked if I wanted a moped. I said no, I do not want that. […] They are good at providing aids. (Senior citizen 14)

However, professionals in particular stressed that the provision of assistive devices was hampered by complicated order systems and extensive delivery time:

We have a user who is completely dependent on a hoist and a wheelchair. When he returned home, he had been assigned an old [and used] wheelchair. All the wheel spokes were destroyed. […] the wheel kept falling off, so that the brakes didn’t work. So it was life [threatening]. It was intended for flat floors, but he had to use it outdoors. (Nurse 18*)*

Usability training was missing, and a lack of information about who to contact for technical support, as well as extensive repair time, further reduced the use of assistive devices. Large and heavy equipment like electronic medicine dispensers, limited senior citizens’ mobility and forced them to stay at home. One senior citizen pointed out:

I didn’t have to wait for them to bring [the medication]. I just had to wait until the machine made the sound. But I couldn’t go out, I had to stay at home at the time that I was supposed to take the medicine. (Senior citizen 7)

Although more expensive devices usually were provided cost free by the municipality, the economic burden of low-cost items such as compression socks, were significant for senior citizens with poor pensions.

Only managers and some senior citizens mentioned reablement as another form of support to enable senior citizens to live longer at home. It could contribute to strengthen their ability to function, and thereby their independence. However, according to managers, time constraints, insufficient focus on what mattered to senior citizens and not letting them make their own decisions, resulted in professionals making decisions on their behalf and in that way taking over the senior citizens’ “tasks”. Instead of reablement, this could result in increased dependency. Moreover, reablement was often offered at a too late stage to fully benefit from its potential:

The municipality should, on their own initiative, intervene at an earlier stage, with a plan for education and training. I know what a stroke is, but I would like to know a little more about which opportunities I have to influence improvements and some more concrete information about how to improve the mobility in my fingers. Physiotherapists often don’t share this information. (Senior citizen 25)

Although senior citizens were asked what mattered to them and provided with choices, managers and professionals pointed out that the number of choices they were given was limited to “this is what we offer” and they were not involved in the decision making processes determining what care they received. Instead, decisions were often solely made by professionals and the municipality, and at times also by carers:

There is no focus on [”what matters to you”]. There are many things that they are not given to decide. For example, they may request that we arrive at 9 am, but we might not arrive before 10 am…We then have to help those who are still in bed first. […] they would have preferred to have it sooner. […] We're probably setting the agenda for what’s okay to bring up. After all, they can’t be demanding when they get help. They just have to be grateful. (Nurse 18)

To better involve senior citizens in their own care, more time should be spent to map their service needs. Senior citizens pointed out that they were not involved in processes to develop and improve the homecare services. In their view, politicians did not know about their home and neighbourhood situation, nor did politicians consult with service user councils. Senior citizens suggested that the municipality should continuously improve the service ecosystem by regularly mapping senior citizens’ needs and service feedback:

A user council for [welfare technology] has not been set up. It would be wise for the municipality to set up such a council. […] continuous improvement is lacking. The municipality standardizes and that’s what we offer. But it might not be well adapted to me and you. […] and needs change over time. (Senior citizen 21)

### Compassionate and competent healthcare professionals

Senior citizens’ descriptions of professionals’ compassion corresponded largely with their perceived ideal, whereas carers and professionals highlighted several limitations to professionals’ compassion. However, senior citizens agreed with carers and managers on aspects of the professionals’ competence which they thought did not correspond with the preferred service ecosystem. Surprisingly, professionals did not focus much on competence as a significant aspect of the existing homecare services (Table 5).

Senior citizens described professionals as friendly, attentive, engaged, caring, and respectful. These professionals took their time, sat down and listened to senior citizens, and engaged with them in conversations. They were perceived as compassionate and able to “see the whole person”. These professionals would regularly ask and check with senior citizens if everything was fine. This was an important part of the care giving process, and professionals provided senior citizens with ample time to respond. They also respected the fact that a private home is different to institutions such as nursing homes. These professionals were also able to differentiate between their own tasks and responsibilities, and those of the senior citizens and carers.

A lack of compassion could on the other hand threaten senior citizens’ dignity and integrity. Some professionals were described as behaving inappropriately and unable to develop a good interpersonal relationship. For example, some professionals did not respect the boundaries of senior citizens’ homes as they made private telephone calls and behaved as if it was their own home. Others were perceived as rude, as they used senior citizens’ toilet without asking for permission or they threw senior citizens’ clothes on the floor and left them there. Some displayed rude behaviour as they spoke to senior citizens in a childlike and patronising manner, gave orders, scolded them or “put them in their place”:

The way they can treat the elderly. Some have been treated like little kids. Some talk to them in a childish manner and may say “go to your room” […] And we have some who speak too harshly. (Skilled healthcare worker 22)

Several examples were given where professionals were in a hurry and rushed through their tasks. Some senior citizens experienced this as being treated in a too harsh manner.

Mostly they are very easy to work with. But some just pull [the corset over the stoma] They are too fast. Yes, they nearly run around here […] Some get annoyed and yell at me. “If you are not happy, then do it yourself”. […] And some people work with their whole body, you feel that they are in a hurry because they do not have much time for each person. (Senior citizen 24)

Others were left on their own for long periods of time, for example hanging in a hoist while professionals made private phone calls. Professionals not attending to their own personal hygiene were described as smelly and physical encounters were unpleasant:

They have little time […] and work so fast that they physically hurt me. […] [They have] bad breath […] and some smell very strongly of perfume or other strong odours. (Senior citizen 25)

Senior citizens and carers offered several possible explanations for why they thought some professionals behaved in ways that suggested that they lacked compassion. Male professionals were described as more relaxed and attentive, compared to female professionals, who were more physically rough:

There are a couple of men here who have a completely different attitude to what they are doing than these running ladies […] [his hand] is paralyzed and when he is stressed, it tenses up so much that the nails can cut through flesh. Or his hand becomes limp. I have had to force his hand up. [The female personnel] have been harsh with him, disrespectful, threw him about and pulled him. Then they have been far too fast. He cannot stand it. They have not taken into account that he needs time to respond to the orders he receives. And he hates to be ordered, that is, impersonal, indifferent, and treated harshly (Carer 2)

Older and more experienced professionals spent more time to get to know each individual senior citizen. They felt treated respectfully by them and “as equals”. Some senior citizens thought that this might be due to professionals having learned “bad manners” as part of their own upbringing. Professionals with insufficient language skills were often less talkative or spoke so loudly that some senior citizens got scared.

Poor leadership could negatively affect the professionals’ compassion. For example, a senior citizen referred to an unpleasant episode of being put in place by a professional during care provision, and thereafter being scolded by this professionals’ manager, when she called the office. She added that the professionals were more compassionate after this manager was replaced. Managers explained that professionals’ ways of expressing compassion (or lack of such) might be influenced by professionals’ own well-being, as well as the continuous struggle to manage time-constraints. For example, some professionals prepared breakfast while senior citizens were at the toilet, thereby resulting in senior citizens feeling loss of control:

[Some professionals try] to use time efficiently when in senior citizens’ homes. […] For example, for a 92-year-old woman, they can act too fast, and she may not have understood what they did. […] She might say that she doesn’t want the professionals to make her bed, but the bed has already been made. (Manager 3)

Due to a feeling of dependence on professionals, some senior citizens and carers tried to win the professionals’ sympathy by collaborating as much as possible, not asking too much, and not complaining nor seeming too demanding.

Nevertheless, some senior citizens, carers and managers described professionals as highly competent and able to support senior citizens well, in line with the preferred service ecosystem. These professionals seemed to possess considerable knowledge and professional insight, and good practical skills. Moreover, they behaved responsibly and were quick at perceiving and responding to senior citizens’ needs. These professionals regularly upgraded their competence through postgraduate training. They appeared to be reflective, flexible, adaptive and found creative solutions to challenges. Moreover, they seemed to be more engaged in and enthusiastic about their work. For example, some visited senior citizens in the hospital. This enabled professionals to better prepare for senior citizens’ return to their homes. These professionals were considered competent to also deal with very sick service users. They had received additional training in medical procedures and technical equipment which was also used in hospital care. They could therefore provide the same type of care at home.

However, most of senior citizens’, carers’ and managers’ descriptions of professionals’ competence presented an entirely different picture, in stark contrast to the preferred service ecosystem. This included the lack of overall competence, experience and training, and for some also insufficient language skills. This applied to some skilled and many unskilled professionals who were described as “having no clue at all”:

We do not have competence to meet all needs, but we try […] Of course we would like to always be competent, but as soon there is more stress, more patients, then one loses the overview, gets stressed and feels incompetent. […] You do not have time to do things properly if you have five assistants who haven’t got a clue. (Manager 5)

These professionals were unable to make important observations of senior citizens. For example, some forgot to provide medication, and they were unable to use assistive devices such as a transfer-slide to move senior citizens from their bed to a wheelchair. Some managers suggested that training on the job was necessary, but senior citizens were not happy about such a practice and found it ineffective:

Some do not have the necessary training to carry out certain tasks. They arrive with a more experienced person. It’s a bit more like “learning by doing” and not always successful. They have to operate machines [hoist], and not always successfully. It’s a combination of tightening belts and starting and stopping the engine with a remote control. You can easily make a mistake that way. (Senior citizen 25)

Managers added that the professionals’ ability to reflect on and to meet senior citizens’ needs was challenged by time-constraints. Professionals were under constant pressure as they were aware of other senior citizens waiting for their assistance. Additionally, many professionals were “task-focused” and seemed to “be on autopilot” implying a culture of “this is how we do it here”. Such behaviour and time-constraints hampered both reflection and the ability to work according to central reablement principles. Moreover, several professionals were unaware that better functioning senior citizens had the potential to function more independently:

I also want [the professionals] to better see the potential of involving senior citizens more actively and not think that as long as it goes well, then that’s good enough. But the senior citizen himself does not always have the prerequisites to be able to either ask for it or see it himself, so professionals need to be able to figure this out by themselves. (Manager 1)

### Continuity of services

All four stakeholder groups highlighted several limitations in the continuity of the existing homecare services. This included both the timeliness and the predictability of services, as well as the (high) number of professionals involved in the care of each senior citizen. This was not in line with the preferred service ecosystem for senior citizens living at home.

The lack of timeliness (punctuality) in the provided homecare services was described by the participants as variations in or changes to the professionals’ time of arrival at the senior citizens’ homes. At times, professionals did not call senior citizens in advance to notify them of their late arrival, and some did not arrive at all. These delays could be due to an unforeseen event requiring professionals to prioritize other service users, staff limitations (e.g., due to sick leave), shift changes, or changes to senior citizens’ care needs. For example, senior citizens’ needs might change considerably as after transferral to or from a nursing home or a hospital. Most commonly, senior citizens had to adapt to schedule changes, rather than the services adapting to meet their care needs. Senior citizens were often unable to choose when they wanted to get out of or into bed, they were required to move appointments with people or services outside their homes to later in the day or to return earlier from social events:

I wouldn’t want to go to bed at 8.30 pm, but that’s when we can provide the services, which I think is sad. Many are therefore forced to live less active lives. We suggest you have a TV in your bedroom, but how nice is it to lie in bed watching TV? And you are actually prevented from participating in social activities. If you live with your wife, going to bed early is a bit undignified. It’s not nice to get a visitor at 9 pm when you need to sit there in your P.J.s. (Manager 5)

Waiting too long for professionals sometimes resulted in undesired situations for senior citizens. Not only could this threaten their dignity, but also further delay the professionals’ schedules:

If I need to go to the toilet and call for help from the homecare services, then I expect them to come quickly, but they do not always. […] If they don’t arrive in time, the stool ends up in the pants. And that’s also more work for them. (Senior citizen 25)

Waiting too long for professionals could also force carers to carry out professionals’ tasks. For example, some senior citizens, such as those living with dementia, became restless and impatient. Professionals were often in a hurry due to time pressures, focusing on a limited number of tasks, and with little or no time for conversations to meet mental health and social needs. Managers stated that professionals were often “putting out fires” rather than implementing reablement programs.

The large number of professionals allocated for each individual senior citizen, in some cases up to 50 per week, resulted in senior citizens having to explain their needs, preferences and routines repeatedly to all the different personnel they encountered. It also prevented professionals from getting to know the senior citizens they provided care for and to build a trusting relationship. In turn, this could negatively impact senior citizens’ sense of safety, especially with activities such as intimate care, and in particular for those living with dementia, anxiety, or psychosocial challenges, and those who were otherwise frail:

It takes so little to “throw off balance” those who are old and frail. You have to repeat everything over and over again and you are never able to develop “good chemistry” with that person […] We have one who is very malnourished and who refuses to receive help with food. What worked was when the same [professional] went to see her over and over again, thereby establishing trust. (Nurse 4).

The large number of professionals working with some senior citizens contributed to a sense of lack of control, discontinuity of information and fragmented care:

Relatives wonder what happens to the information when asking about medication […] The answer is then: I do not know, I have not been here before […] no continuity. Carers are worried that someone will make a mistake because there are so many professionals. […] There are so many people passing by, one does not know what the other is doing. (Manager 1)

Discontinuity of services can also increase the number of adverse events, including mistakes with medication and infection control (e.g., during COVID-19). Instead of alleviating the carers’ burden, the high number of professionals providing care for a single senior citizen may add to it if they are summoned frequently, for example to provide practical information. Professionals described this as frustrating, depressing, and an energy drain for senior citizens. Mostly it is unpredictable for those who would turn up, and professionals execute their tasks in different ways. Managers explained that the high number of different professionals for individual senior citizens was due to the use of different professionals during night shifts and in the weekends, as well as during summer holidays and due to staff sick leave.

Having said this, some senior citizens were indifferent to the large number of professionals they had to relate to and some appreciated meeting a variety of professionals with different cultural backgrounds. Nevertheless, senior citizens found it was easier to remember or get to know professionals from the reablement team due to its limited size. They also established better relationships with their primary contact or those professionals who sat down for a conversation and thereby indicated that they had time for them:

It can be tiring with so many different [professionals]. It was tiring, always a new one, almost always. Not to complain about them, but I guess they cannot do it another way. We never get to know them. […] I know my [primary] contact, but she’s rarely there. Usually there are others. (Senior citizen 5)

### Accessible services and information

Contrary to carers, the other stakeholders found that access to free services 24/7 corresponded with the preferred service ecosystem. According to them, response time in the event of an emergency was mostly good, although examples of late response were also described. Finding the right information (e.g., about assistive devices) and the responsible persons or institutions was often time consuming and challenging. For example, contact with public services responsible for distribution of senior citizens’ assistive devices or pensions, was described as bureaucratic and difficult:

The general problem with the municipality: you get nothing unless you go and knock on a door. My wife and I have discovered how to get information, but this is very tiring for her […] there is insufficient information about the service options. (Senior citizen 25)

A carer added that she received ambiguous answers: “It was awful, one week we got one answer, the next week another. First that you cannot and then a letter to say [the opposite].” Managers explained that this was due to a complex service organisation characterised by «silo operations» and budget restrictions. This negatively influenced the collaboration, coordination and information flow between the different municipal departments. A complex service organisation limited the flow of service provision for senior citizens.

The access to senior citizen centres was in line with the preferred service ecosystem, as these centres met senior citizens’ psychosocial needs and reduced carer burden. Surprisingly, carers themselves did not emphasise the importance of senior citizen centres (Table 5). Public taxi-buses facilitated transport to these centres. However, more flexible and longer opening hours was requested, as it would alleviate carers further, as well as providing an option to better adapt the availability of senior citizen centres to their other daily activities and biological rhythm:

For some senior citizens it is too early. If you live alone and you have cognitive impairment, then stay up all night to get ready and to keep track of when they are going to the senior citizens centre, when will the bus arrive? Or is it the right day? it creates unrest. You can have a clear head, but being ready at 8:30 am, when the bus arrives, that’s a burden. Being picked up later would help doing things at their own pace. (Manager 4)

Senior citizens and professionals suggested senior citizen centres should offer a greater variety of entertainment, including some that would encourage senior citizens’ active participation:

We have such good service, we are being served all day long, we do not have to lift a finger. But days can become long sometimes. There is little entertainment. We're just sitting there. (Senior citizen 5).

Moreover, the service offers at senior citizen centre should be regularly assessed and adapted to be in line with the rest of society and to better meet the changing needs of the population of senior citizens:

Another [senior citizen] who would really benefit mentally from being with people, but sitting in a daycare center and maybe doing the “head, shoulders, knees and toes” song. Give me a place where I can paint and use my creativity. So adapt activities to different types of personalities. Not just […] prayer and bingo…(Nurse 18)

## Discussion

In order to develop the preferred service ecosystem for senior citizens living at home, this study applied an idealized design approach. The study aimed to understand how stakeholders found that the existing homecare services corresponded with or differed from the preferred service ecosystem. This is the first study assessing this from a multiple stakeholder perspective, by bringing together senior citizens’, carers’, healthcare professionals’, and managers’ views on the existing homecare services.

All stakeholders emphasised challenges with the professionals’ competence and continuity of services. Contrary to other stakeholders, the senior citizens’ views of the existing support for living at home as long as possible corresponded to a large extent with their preferred service ecosystem. Time, resources and a complex municipal organisation significantly impacted all aspects of the existing homecare services.

The existing research literature suggests that the preferred service ecosystem should support senior citizens’ self-determination and social participation, and enable them to live at home as long as possible (2, 23, 44). The service ecosystem should be predictable, adaptive, facilitate relationships, and continuously involve senior citizens in its development and in service provision. We discuss these issues below, in light of the current study results (summarized in Table 6).

**Table 6:** Development of the preferred service ecosystem.

Aspect	Preserve	Improve/redesign
General	** **	Time, resources, and service ecosystem design
Predictable and adaptive service ecosystem	Extensive practical support	Increase nursing home capacity
	Social contact through home visits	
	24/7 service accessibility	
	User-friendly assistive devices	A simplified order system: enhance availability; safeguard prompt delivery of user-friendly assistive devices
	Municipal recommendations emphasise WMTY^a^ and reablement	Ensure flexibility in accommodating WMTY^a^ and facilitating self-determination
	Competent healthcare professionals	Ensure competent and reflective professionals
	Accessibility of senior citizen centres	Extend opening hours and improve service provision at senior citizen centres
		Ensure punctuality
		Provide unambiguous information
		Alleviate carer burden
		Safeguard nutrition
		Ensure accessible public transport
Relationships	Compassionate professionals	Provide compassionate professionals
		Limit the number of professionals
Involvement		Ensure effective involvement in service delivery
		Improve involvement in service ecosystem co-design

^a^ What matters to you.

### Predictable and adaptive service ecosystem

The existing homecare services are freely accessible 24/7 and provide extensive practical and social support, e.g., through professionals’ home visits. Both reablement and assistive devices support senior citizens to live at home as long as possible. Improvement includes aspects such as better accommodating “What Matters To You”-questions [WMTY], limiting carer burden, improving punctuality, simplification of the order system of assistive devices, strengthening professionals’ competence, and providing predictable and accessible public transport (Table 6).

Previous studies, reporting on single aspects of the homecare services or derived from a single stakeholder group, support our findings, including professionals asking WMTY-questions, and providing extensive practical support (45, 46). Currently, senior citizens must adapt to an unpredictable and predetermined service ecosystem, including professionals’ working routines (45, 47), inaccessible public transport (48–50), and incompetent professionals (51, 52). As a consequence, senior citizens risk becoming “*guests*” in their own homes (1, 53, 54).

Aspects of professionals’ competence addressed in previous research has been how professionals may struggle with asking WMTY, which requires sufficient time, proper responses from senior citizens, and value in practice (46). To our surprise, the professionals themselves emphasised the significance of reablement and their own competence to a limited extent. This may align with competence being generally poorly identified and defined in the service ecosystem for senior citizens (55).

Though previous research on the impact of punctuality on senior citizens’ lives is limited, some studies confirm the importance of fast response to urgent needs, as well as the importance of senior citizens being informed about possible delays in service delivery (20, 54, 56).

### Relationships

The results of our study suggest that within the existing homecare services some professionals are experienced as compassionate, whereas others are not. Yet, compassion is a significant requirement, and mutual and trusting relationships should be facilitated by a suitable number of professionals. Furthermore, some senior citizens may feel more comfortable with the close relationship that is required within the homecare services if professionals also pay attention to their own personal hygiene.

Several studies confirm the need for compassionate professionals and limiting the number of professionals senior citizens must relate to (10, 20, 54, 57). In contrast to this, our findings and previous studies confirm that a few senior citizens were not concerned about the number of professionals, but found it interesting to meet new people, as long as they were skilled and known to the senior citizens (45, 58, 59).

While senior citizens value social inclusion and respect within the community (7, 60, 61), some may experience loneliness and perceive professionals as friends or family members (45, 62, 63). At the same time, they may sense the professionals’ lack of time and be reluctant to burden them (1, 53, 64). This requires professionals to be both sensitive to senior citizens’ needs, as well as competent in supportive communication (53, 65). However, some participants in our study stated that senior citizens’ dignity and integrity were at stake due to professionals who could be harsh or rude. Similar findings have been found in other studies (47). Some senior citizens chose their words with caution or refrained from stating their opinion to avoid damaging their relationships with the professionals. This cautious approach suggests power differentials (14, 66, 67). Several explanations were provided to explain why some professionals were compassionate and others not, including gender, age, and work experience. Others suggest professionals lacking emotional competence and ability to balance senior citizens’ needs with routines (68, 69). Our findings suggest a task-based rather than person-based focus. Previous studies about the impact of professionals’ own personal hygiene on the relationships and caregiving process were not found, which our study adds to.

### Involvement

Our results suggest that improvements are needed for senior citizens’ involvement in both service delivery and the development of the service ecosystem. Although WMTY was asked, senior citizens were still offered a limited number of service options. Hence, their opportunities for self-determination were limited. As an explanation, lack of time and resources was reiterated. Additionally, the organisation of the existing homecare services was perceived as complex, therewith challenging e.g., collaboration, coordination, and information flow across the various municipal departments. Apparently, municipal decisionmakers did not involve senior citizens in organisational decisions.

Lack of involvement at both an individual and organisational level is supported by previous studies, confirming challenges to integrate it into the existing homecare services (13, 46, 70, 71). Previous research also suggests that stakeholder involvement and WMTY should be both the starting point and the continuum in the development of the preferred service ecosystem (31, 72). At the same time, our findings illustrate the complexity of how all characteristics of the service ecosystem are intertwined and interconnected. Consequently, research suggests that an (eco)system thinking approach should guide a co-design process to succeed in developing the preferred service ecosystem (15, 23, 31, 72–74).

### Strengths and limitations

This study was unique in including the perspectives of four stakeholder groups on the existing homecare services and how they corresponded with the preferred service ecosystem for senior citizens living at home. The four-category framework developed in the first stage of the research project (2) was applied in the second stage, in line with an idealized design approach (34–36). A limitation of this is that the same participants contributed in the two stages. However, testing of the framework in the second stage added time, resources and organisation as important aspects of the preferred service ecosystem. Although another limitation might be that certain stakeholder groups, such as untrained assistants, were not involved, the study included multiple perspectives provided by senior citizens, carers, healthcare professionals, and managers. It is our understanding that both individual and focus group interviews provided in depth understanding of the different stakeholders’ experiences and perspectives. However, one interview approach was more suitable to some participants, and vice versa. Furthermore, additional individual or focus group interviews might further expand different stakeholders’ perspectives. Other data collection methods could have been considered to further explore the perspectives of senior citizens who had some difficulties in expressing themselves, whether due to age or health challenges. Whereas the preferred service ecosystem might include WHO's age-friendly domains such as social participation and inclusion (2, 24, 75), the participants provided limited perspectives on these domains. Even though the majority of current research studies report findings from senior citizens’ perspectives only, they correspond with the multiple stakeholder perspectives in our study.

### Implications

The current study has various implications for practice. It confirms that the involvement of multiple stakeholders may contribute to perspectives which can be used to transforming the existing homecare services. The results suggest that in particular, the continuity of services and healthcare professionals’ competence should be improved. The latter may require changes in healthcare education, recruitment policies and supervision structures. Furthermore, age-friendly infrastructures such as access to flexible and better adapted public transport are needed. User-friendly assistive devices should be provided promptly, and order systems should be simplified. The providers of senior citizens centres should consider a broader range of service provision.

The current study has important implications for further research. The rationale for and impact of professionals’ limited focus on their overall skills and the need for reablement competence in particular should be investigated. Additionally, there is a need for further knowledge about how power differentials may be reduced, how senior citizens’ dependency on professionals may be limited, and how senior citizens can best be involved in their own care and in further developing the services.

The four-category framework should be further tested to assess its usefulness, if and how it should be improved, as well as to assess its transferability to other healthcare contexts such as nursing homes and hospital care.

## Conclusion

This study found that certain aspects of the existing homecare services corresponded with the preferred service ecosystem according to four stakeholder groups in a Norwegian municipality, whereas other aspects of the existing services differed considerably from the idealized design. The stakeholders agreed that there was a need to improve healthcare professionals’ competence and the continuity of services. Senior citizens were satisfied with the practical and social support of the homecare services, while carers, healthcare professionals, and managers elaborated on specific challenges with the support services. To develop the preferred service ecosystem, aspects such as predictability, adaptivity, relationships, and continuous involvement of senior citizens are key. The four-category framework applied in this study could support the development of the preferred service ecosystem provided that essential aspects such as time, resources and organisation are being integrated.

## Data Availability

The raw data supporting the conclusions of this article will be made available by the authors, without undue reservation.
