# Supplementary material for: How do the existing homecare services correspond with the preferred service ecosystem for senior citizens living at home? A qualitative interview study with multiple stakeholders
Source: Front Health Serv. 2024 Mar 21;4:1294320. doi: 10.3389/frhs.2024.1294320 (PMC10991764; doi:10.3389/frhs.2024.1294320)
Supplement: Supplementary file 1 [file Table1.pdf]

Supplementary Table 1: example of the analysis process (six stages)

Stage 2	***Stage 3***	***Stage 4***	***Stage 5***		***Stage 6***[1]	
**Transcript**	**Condensed meaning unit**	**Code**	**Sub-category**	**Category**	**YES**	**NO**
[...] And his clothes were thrown on the floor when they left. This is not a nursing home… it is a private living room… .it is our private home. [...] I said «please be so kind and arrive earlier because my husband has problems, he is upset because he had to sit and wait for almost an hour… Can you not pay some attention to him?»… [...] he needs to get to the toilet. then I saw the mess they left behind…… We always manage to keep it up, unless we don't have a bad stomach… preferably a quarter of an hour, preferably twenty minutes, but we can't manage a whole hour. It is not worthy… is what I said….Then I felt «that day I have said enough». [...]	his clothes were thrown on the floor when they left	clothes thrown on the floor	Professionals' compassion	Compassionate and competent healthcare professionals		X
	he was upset [because he had to wait for almost one hour, toilet visit]	upset because of waiting time toilet visit	Timeliness and predictability	Continuity of services		X
	he had to sit and wait for almost one hour (toilet visit)	waiting for one hour (toilet visit)	Timeliness and predictability	Continuity of services		X
	The mess they left behind [clothes on the floor]	mess/clothes on the floor	Professionals' compassion	Compassionate and competent healthcare professionals		X
	Unworthy with one hour waiting time (toilet visit)	1 hour waiting (toilet visit) unworthy	Timeliness and predictability	Continuity of services		X

[1]Correspondence with the preferred service ecosystem
